# *CDH12* as a Candidate Gene for Kidney Injury in Posterior Urethral Valve Cases: A Genome-wide Association Study Among Patients with Obstructive Uropathies

**DOI:** 10.1016/j.euros.2021.04.001

**Published:** 2021-04-24

**Authors:** Loes F.M. van der Zanden, Iris A.L.M. van Rooij, Josine S.L.T. Quaedackers, Rien J.M. Nijman, Martijn Steffens, Liesbeth L.L. de Wall, Ernie M.H.F. Bongers, Franz Schaefer, Marietta Kirchner, Rouven Behnisch, Aysun K. Bayazit, Salim Caliskan, Lukasz Obrycki, Giovanni Montini, Ali Duzova, Matthias Wuttke, Rachel Jennings, Neil A. Hanley, Natalie J. Milmoe, Paul J.D. Winyard, Kirsten Y. Renkema, Michiel F. Schreuder, Nel Roeleveld, Wout F.J. Feitz

**Affiliations:** a Radboud Institute for Health Sciences, Department for Health Evidence, Radboud university medical center, Nijmegen, The Netherlands; b Department of Urology, University Medical Center Groningen, Groningen, The Netherlands; c Department of Urology, Isala, Zwolle, The Netherlands; d Radboud Institute for Molecular Life Sciences, Division of Pediatric Urology, Department of Urology, Radboudumc Amalia Children’s Hospital, Nijmegen, The Netherlands; e Radboud Institute for Molecular Life Sciences, Department of Human Genetics, Radboud university medical center, Nijmegen, The Netherlands; f Center for Pediatrics and Adolescent Medicine, University Hospital Heidelberg, Heidelberg, Germany; g Institute of Medical Biometry and Informatics, University of Heidelberg, Heidelberg, Germany; h Department of Pediatric Nephrology, Faculty of Medicine, Cukurova University, Adana, Turkey; i Department of Pediatric Nephrology, Istanbul University-Cerrahpasa, Istanbul, Turkey; j Department of Nephrology, Kidney Transplantation and Hypertension, Children’s Memorial Health Institute, Warsaw, Poland; k Pediatric Nephrology, Dialysis and Transplant Unit, Fondazione IRCCS Ca’ Granda, Ospedale Maggiore Policlinico di Milano, Milan, Italy; l Department of Clinical Sciences and Community Health, University of Milan, Milan, Italy; m Division of Pediatric Nephrology, Hacettepe University Faculty of Medicine, Ankara, Turkey; n Institute of Genetic Epidemiology, Faculty of Medicine and Medical Center, University of Freiburg, Freiburg, Germany; o Faculty of Biology, Medicine and Health, Manchester Academic Health Sciences Centre, University of Manchester, Manchester, UK; p Endocrinology Department, Manchester University NHS Foundation Trust, Manchester, UK; q Nephro-Urology Research Group, Developmental Biology and Cancer Programme, UCL Great Ormond Street Institute of Child Health, London, UK; r Department of Genetics, Center for Molecular Medicine, University Medical Center Utrecht, Utrecht University, Utrecht, The Netherlands; s Radboud Institute for Molecular Life Sciences, Department of Pediatric Nephrology, Radboudumc Amalia Children’s Hospital, Nijmegen, The Netherlands

**Keywords:** CDH12, Genome-wide association study, Obstructive uropathy, Posterior urethral valves

## Abstract

**Background:**

Posterior urethral valves (PUVs) and ureteropelvic junction obstruction (UPJO) are congenital obstructive uropathies that may impair kidney development.

**Objective:**

To identify genetic variants associated with kidney injury in patients with obstructive uropathy.

**Design, setting, and participants:**

We included 487 patients born in 1981 or later who underwent pyeloplasty or valve resection before 18 yr of age in the discovery phase, 102 PUV patients in a first replication phase, and 102 in a second replication phase.

**Outcome measurements and statistical analysis:**

Signs of kidney injury were defined as dialysis, nephrectomy, kidney transplantation, estimated glomerular filtration rate (eGFR) <60 ml/min/1.73 m^2^, high blood pressure, antihypertensive medication use, proteinuria, and/or one kidney functioning at <45%. We used χ^2^ tests to calculate *p* values and odds ratios for >600 000 single-nucleotide polymorphisms (SNPs) in the discovery sample comparing patients with and without signs of kidney injury within 5 yr after surgery. We performed stratified analyses for PUV and UPJO and Kaplan-Meier and Cox regression analyses in the discovery and two replication samples for the associated SNPs, and RNA and protein expression analyses for the associated gene in fetal tissues.

**Results and limitations:**

Despite the small and nonhomogeneous sample, we observed suggestive associations for six SNPs in three loci, of which rs6874819 in the *CDH12* gene was the most clear (*p* = 7.5 × 10^–7^). This SNP also seemed to be associated with time to kidney injury in the PUV discovery and replication samples. RNA expression analyses showed clear *CDH12* expression in fetal kidneys, which was confirmed by protein immunolocalization.

**Conclusions:**

This study identified *CDH12* as a candidate gene for kidney injury in PUV.

**Patient summary:**

We found that variants of the *CDH12* gene increase the risk of kidney injury in patients with extra flaps of tissue in the urethra (posterior urethral valves). This is the first report on this gene in this context. Our study provides interesting new information about the pathways involved and important leads for further research for this condition.

## Take Home Message

We found that variants of *CDH12* increase the risk of kidney injury in patients with posterior urethral valves. This is the first time that this gene has been reported on in this context. Our study provides interesting new information about pathways involved in obstructive uropathies and important leads for further research.

## 1 Introduction

Posterior urethral valves (PUV) and ureteropelvic junction obstruction (UPJO) are congenital anomalies of the urinary tract that impair urinary flow. PUV affects only boys and occurs in one in 4000 live male births [1], while UPJO affects one in 500 children [2]. These obstructions may perturb kidney development and are referred to as obstructive uropathy [3]. Obstructive uropathy is the second most prevalent cause of end-stage kidney disease in children [4], but long-term kidney function is variable and cannot be predicted reliably.

Knowledge about the molecular pathways involved in the pathophysiology of kidney injury in congenital obstructive uropathy has mainly been derived from neonatal rats and mice with induced complete unilateral ureteral obstruction (UUO). Kidney response to experimental UUO involves oxidative stress, monocyte infiltration, uncontrolled apoptosis of tubular cells, fibroblast accumulation, and increased deposition of extracellular matrix [3]. This is similar to the response in human obstructed kidneys, where the extent of monocyte infiltration correlates with the intensity of tubulointerstitial damage, and inflammatory molecules, such as MCP1, are upregulated [5]. In addition to elements from proinflammatory pathways, several other molecules have been suggested to play a pathological role in kidney injury in obstructive uropathy in animal models (such as angiotensin II and Tgf-β) [3], [6], [7]. Several of these were confirmed in human expression studies (including TGF-β1, REN, and EGF) [5], [6], [8], [9]. Finally, copy number variations (CNVs) >100 Kb were associated with kidney failure in PUV patients [10], while polymorphisms in ACE and AGTR2 have been associated with lower estimated glomerular filtration rate (eGFR), chronic kidney disease, and kidney scarring in PUV patients [11], [12], [13].

Most of the genetic studies described above focused on candidate genes, but a hypothesis-generating approach, such as a genome-wide association study (GWAS), may shed new light on pathways involved in the development of kidney injury in obstructive uropathy. In addition, genetic variants associated with kidney function decline could be used in prognostic modeling approaches to better predict long-term kidney function. As no such studies have been conducted so far, the aim of this study was to perform a GWAS for kidney injury in obstructive uropathy. We included patients with PUV and UPJO under the assumption that similar genetic variants are associated with kidney function decline in both obstructive uropathies.

## 2 Patients and methods

### 2.1 Patients

#### 2.1.1 Discovery sample

AGORA (Aetiologic research into Genetic and Occupational/environmental Risk factors for Anomalies in children) is a database and biobank in the Radboudumc (Nijmegen, The Netherlands) collecting questionnaire data, blood/saliva samples, and phenotypic information from patients with congenital malformations and their parents [14], [15]. We identified patients born from 1981 onwards who underwent pyeloplasty or PUV resection before 18 yr of age in AGORA and searched the Radboudumc medical registry for additional patients to approach for participation.

#### 2.1.2 Dutch replication sample

For replication purposes, we included patients treated in the Isala clinics, Zwolle or University Medical Center (UMC), Groningen, The Netherlands. Owing to registration issues, we were only able to identify patients treated in 2002 or later.

#### 2.1.3 European replication sample

The European replication sample was derived from the 4C study [16]. In brief, a cohort of children aged 6–17 yr with chronic kidney disease (eGFR 10–45 ml/min/1.73 m^2^) was enrolled between 2010 and 2012 and followed until 2018 at 39 pediatric nephrology centers in eight European countries. We selected children with PUV as the underlying diagnosis for whom genotype data, longitudinal eGFR measurements, and information on treatment changes were available.

### 2.2 Kidney injury

The medical files for the Dutch patients were scrutinized for information about kidney function and for clinical descriptions. Patients were defined as having signs of kidney injury in case of dialysis, nephrectomy, kidney transplantation, eGFR <60 ml/min/1.73 m^2^, high blood pressure, antihypertensive medication use, proteinuria, and/or one kidney functioning at <45%. In the European replication sample, the primary endpoint was kidney injury defined as a composite of 50% loss of eGFR, eGFR <10 ml/min/1.73 m², or kidney replacement therapy (Supplementary material).

### 2.3 Genotyping, quality control, and imputation

The Dutch samples were genotyped by deCODE Genetics (Reykjavik, Iceland) using Infinium OmniExpress bead chips or Global Screening arrays (Illumina, San Diego, CA, USA). European replication samples were genotyped using Illumina Infinium 2.5M-8 microarrays. Quality control and imputation are described in the Supplementary material.

### 2.4 Statistical analysis

We excluded patients with <5 yr of follow-up from our discovery sample and compared patients with and without signs of kidney injury within 5 yr after surgery using the PLINK whole-genome data analysis toolset (www.cog-genomics.org/plink/1.9/). Analyses were adjusted for the first four principal components to account for population stratification. We repeated the analyses with imputed genotypes for the area showing an association signal and visualized the results in LocusZoom (http://locuszoom.org/). For single-nucleotide polymorphisms (SNPs) passing the threshold of *p* = 1 × 10^–5^, we examined the associations separately for PUV and UPJO patients to verify the assumption of similar genetic variants associated with kidney function decline in these phenotypes, performed Kaplan-Meier and Cox regression analyses to include 130 patients with <5 yr of follow-up, and checked results in the replication samples. In additional analyses, we adjusted the Cox regression analysis for the most significant SNP in the PUV subgroup of the discovery sample for the possible prognostic factors from Table 1. In addition, we performed Cox regression analyses for the allelic effect of this SNP in the discovery and both replication samples, and combined the results in a meta-analysis in Review Manager 5 (https://training.cochrane.org/online-learning/core-software-cochrane-reviews/revman/revman-5-download) using the inverse-variance method and a random-effects model.

**Table 1:** Characteristics of the patient population used in the discovery analyses

	Patients, *n* (%)	
	PUV group (*N* = 316)	UPJO group (*N* = 171)
Antenatal detection of urinary tract abnormalities	68 (22)	77 (45)
Birth weight <2500 g	19 (6)	14 (8)
Hospital admission within 1 wk after birth for at least 7 d	60 (19)	26 (15)
Febrile urinary tract infections in the first 4 yr of life a	108 (34)	34 (20)
Kidney abnormalities on ultrasound b	31 (10)	47 (28)
Bladder dysfunction c	130 (41)	6 (4)
Age at pyeloplasty or PUV resection		
<1 yr	124 (39)	51 (30)
1–4 yr	59 (19)	49 (29)
5–12 yr	130 (41)	60 (35)
13–17 yr	3 (1)	11 (6)
Signs of kidney injury d	93 (29)	90 (53)
Dialysis, nephrectomy or kidney transplantation	17 (5)	5 (3)
eGFR <60 ml/min/1.73 m^2^	34 (11)	11 (6)
High blood pressure or antihypertensive medication	31 (10)	22 (13)
Proteinuria	11 (3)	6 (4)
One kidney functioning < 45%	55 (17)	74 (43)

PUV = posterior urethral valve; UPJO = ureteropelvic junction obstruction; eGFR = estimated glomerular filtration rate.

a Urinary tract infections after surgery were not taken into account.

b Kidney abnormalities defined as the presence of kidney scars or cysts, kidney dysplasia, increased echogenicity, and/or loss of parenchyma.

c Bladder dysfunction defined as training or use of medication to improve bladder function, intermittent catheterization, or vesicostoma/ureterocutaneostoma.

d Signs of kidney injury developed during the maximum follow-up period of 10 yr after surgery. Some patients developed more than one of these signs, which is why the numbers do not add up to the total number of patients with signs of kidney injury.

### 2.5 Expression analyses

We used the Expression Atlas (www.ebi.ac.uk/gxa/home) in February 2019 to see which tissues *CDH12* is expressed in [17]. We tested expression in adult human kidney and brain and in commercially available mRNA from human fetal kidneys (Clontech Laboratories, Mountain View, CA, USA) via quantitative polymerase chain reaction. We used data from our transcriptomic analysis of human organogenesis covering 15 embryonic tissues and organs 33–55 d after conception [18] to identify embryonic tissues in which the gene is expressed.

We used the Protein Atlas (www.proteinatlas.org/) in February 2019 [19] and performed immunohistochemistry of human fetal tissues as previously described [20], using a primary antibody to CDH12 (ab71055; Abcam, Cambridge, UK) at a dilution of 1:100. Tissue processing was as previously described [21].

Finally, we checked the GTEx portal (https://gtexportal.org/home/) in February 2019 to see whether the SNPs that we identified are expression quantitative trait loci (eQTL) influencing expression of *CDH12*.

## 3 Results

### 3.1 Patients

#### 3.1.1 Discovery sample

AGORA contained DNA from 433 eligible patients. We identified 247 additional patients, of whom 151 (61%) participated (Supplementary Fig. 1). We excluded 40 patients who did not have their first surgery (pyeloplasty or PUV resection) in the Radboudumc, four patients with end-stage kidney disease before surgery, and five patients without follow-up information. Of the remaining 535 samples, 12 failed genotyping and 36 failed sample quality control (one sample with a call rate <97%, one discordant sex information, four related individuals, and 30 ethnic outliers; Supplementary Fig. 2). Genotyping information for 487 patients was available for the discovery analyses (Table 1).

#### 3.1.2 Dutch replication sample

For replication purposes, we identified 131 patients treated in the Isala clinics and 270 in UMC Groningen. Of these patients, 87 (66%) and 164 (61%), respectively, participated. We excluded three patients without follow-up information and 26 samples that failed quality control (two samples with a call rate <97%, one related individual, and 23 ethnic outliers; Supplementary Fig. 2). We used genotyping information for 222 patients in the replication analyses.

#### 3.1.3 European replication sample

The European replication sample was derived from a study including 704 children [16]. PUV was the underlying diagnosis for 119 children, and all necessary information was available for 102 of these.

### 3.2 Discovery analyses

**Fig. 1:**
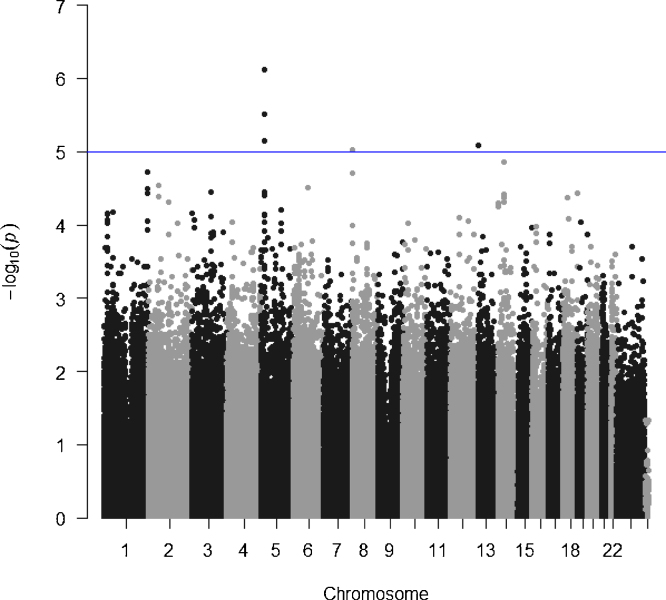
Manhattan plot of genome-wide association study results for 141 PUV and UPJO patients with signs of kidney injury 5 yr after surgery and 216 PUV and UPJO patients without signs of kidney injury after 5 yr. The blue line represents the threshold for suggestive genome-wide significance (*p* < 1 × 10^–5^). The six single-nucleotide polymorphisms above this threshold are rs6874819 (*p* = 7.5 × 10^–7^), rs9292998 (*p* = 3.1 × 10^–6^), and rs12171538 (*p* = 7.1 × 10^–6^) on chromosome 5, rs2957086 (*p* = 9.3 × 10^–6^) on chromosome 8, and rs9580025 (*p* = 8.2 × 10^–6^) and rs2148707 (*p* = 8.2 × 10^–6^) on chromosome 13.

We excluded 130 patients with <5 yr of follow-up and compared 141 patients with and 216 patients without signs of kidney injury within 5 yr after surgery. Six SNPs in three loci reached suggestive genome-wide significance (p < 1 × 10^–5^; Fig. 1 and Supplementary Table 1), with rs6874819 on chromosome 5 being the most significant SNP (*p* = 7.5 × 10^–7^). The other signals were from chromosomes 8 (rs2957086; *p* = 9.3 × 10^–6^) and 13 (rs9580025 and rs2148707; both *p* = 8.2 × 10^–6^). Genotype frequencies were in Hardy-Weinberg equilibrium in controls (*p* values 0.23–0.95), except for rs2957086 (*p* = 0.04). After imputation, rs6874819 remained the SNP with the strongest signal.

Subgroup analyses for the six SNPs in the PUV (*n* = 235) and UPJO (*n* = 122) groups separately showed that the signals on chromosomes 5 and 13 were stronger in the PUV group, while the signal on chromosome 8 was stronger in the UPJO group (Supplementary Table 2).

**Fig. 2:**
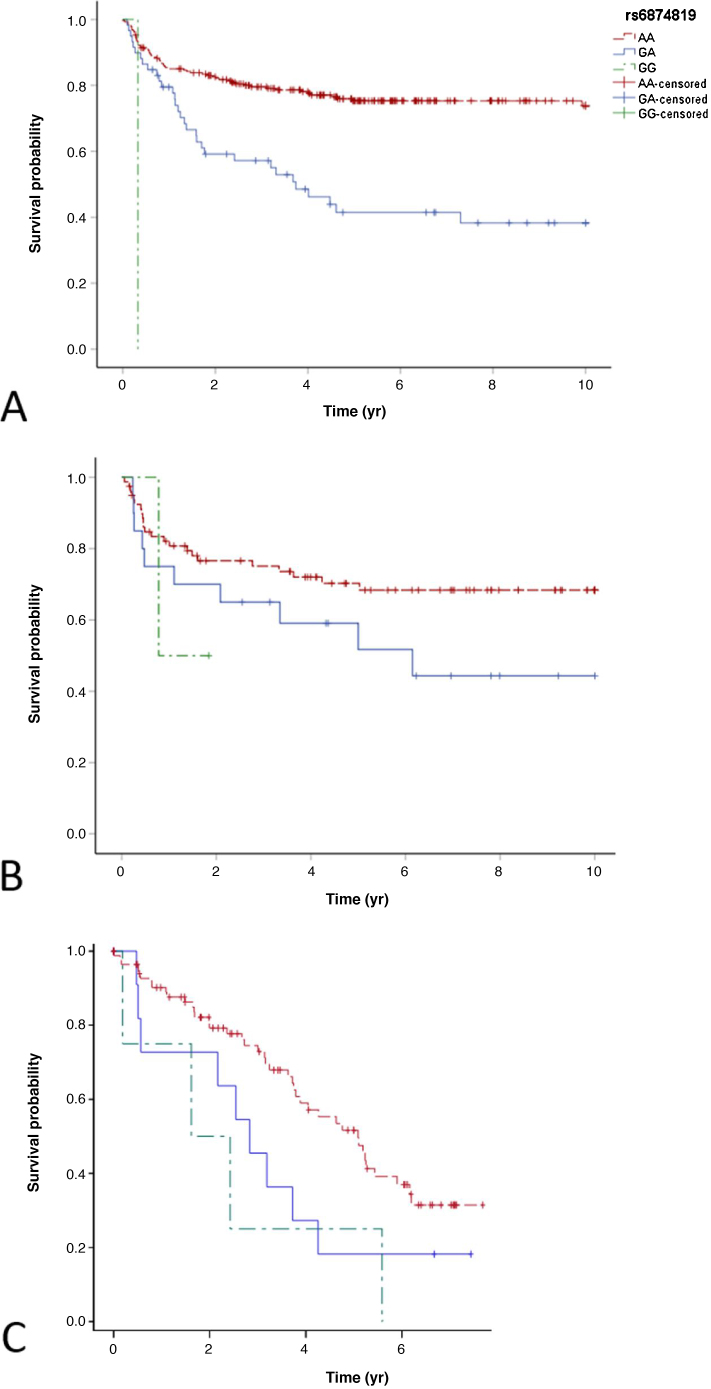
Kaplan-Meier plots showing the probability of surviving without kidney injury for PUV patients by rs6874819 genotype in (A) the Dutch discovery sample, (B) the Dutch replication sample, and (C) the European replication sample.

Kaplan-Meier and Cox regression analyses were performed to include 130 patients with <5 yr of follow-up. These analyses included 316 PUV and 171 UPJO patients, of whom 93 and 90, respectively, developed signs of kidney injury within 10 yr after surgery. Variant genotypes of the SNPs on chromosomes 5 and 13 were statistically significantly associated with time to the development of signs of kidney injury among PUV patients (Fig. 2A for rs6874819), whereas the results for UPJO patients were less consistent (Table 2).

**Table 2:** HRs for kidney injury for SNPs passing the 1 × 10^−5^ threshold separately for PUV and UPJO patients in the Dutch discovery and replication samples, and for SNPs on chromosome 5 in the European replication sample

Chromosome and SNP	Dutch discovery sample				Dutch replication sample				European replication sample	
	PUV patients		UPJO patients		PUV patients		UPJO patients		PUV patients	
	*N* = 316 (93 events)		*N* = 171 (90 events)		*N* = 102 (34 events)		*N* = 120 (65 events)		*N* = 102 (54 events)	
	HR (95% CI)	*p* value	HR (95% CI)	*p* value	HR (95% CI)	*p* value	HR (95% CI)	*p* value	HR (95% CI)	*p* value
**Chromosome 5**										
rs6874819										
AA genotype	Reference		Reference		Reference		Reference		Reference	
GA genotype	2.8 (1.8–4.3)	3 × 10^−6^	2.4 (1.5–3.8)	1 × 10^−3^	1.8 (0.9–3.8)	0.11	1.1 (0.5–2.2)	0.84	1.8 (0.9–3.9)	0.11
GG genotype	18 (2.4–131)	5 × 10^−3^	3.0 (0.7–13)	0.12	2.4 (0.3–18)	0.40	–	–	3.1 (1.1–9.1)	0.04
rs9292998 a										
AA genotype	Reference		Reference		Reference		Reference		Reference	
GA genotype	2.7 (1.7–4.1)	8 × 10^−6^	2.4 (1.4–4.0)	1 × 10^−3^	1.6 (0.7–3.5)	0.22	1.1 (0.5–2.2)	0.84	1.7 (0.8–3.8)	0.17
GG genotype	17 (2.3–127)	6 × 10^−3^	3.0 (0.7–12)	0.13	2.3 (0.3–17)	0.42	–	–	3.1 (1.1–9.0)	0.04
rs12171538 a										
TT genotype	Reference		Reference		Reference		Reference		Reference	
CT genotype	2.1 (1.4–3.3)	1 × 10^−3^	1.4 (0.9–2.3)	0.13	0.9 (0.4–1.9)	0.80	1.6 (1.0–2.8)	0.08	1.0 (0.5–1.9)	0.89
CC genotype	5.8 (2.9–12)	5 × 10^−7^	1.5 (0.5–4.0)	0.46	3.8 (0.9–16)	0.08	0.5 (0.1–3.6)	0.49	3.1 (1.3–7.5)	0.01
**Chromosome 8**										
rs2957086^a^										
AA genotype	Reference		Reference		Reference		Reference			
GA genotype	1.0 (0.6–1.7)	1.0	1.2 (0.7–2.0)	0.56	1.0 (0.4–2.1)	0.91	1.2 (0.7–2.1)	0.50		
GG genotype	2.3 (1.3–4.1)	3 × 10^−3^	2.5 (1.4–4.3)	1 × 10^−3^	0.7 (0.2–1.9)	0.46	0.9 (0.4–1.9)	0.78		
**Chromosome 18**										
rs9580025 a										
CC genotype	Reference		Reference		Reference		Reference			
TC genotype	2.0 (1.3–3.0)	2 × 10^−3^	1.3 (0.8–2.0)	0.30	1.1 (0.5–2.1)	0.82	0.8 (0.5–1.4)	0.45		
TT genotype	2.7 (1.3–5.5)	8 × 10^−3^	3.8 (1.8–8.2)	1 × 10^−3^	–	–	1.1 (0.4–3.0)	0.90		
rs2148707										
GG	Reference		Reference		Reference		Reference			
TG	2.0 (1.3–3.0)	2 × 10^−3^	1.3 (0.8–2.0)	0.30	1.1 (0.5–2.1)	0.82	0.9 (0.5–1.4)	0.59		
TT	2.7 (1.3–5.5)	8 × 10^−3^	3.8 (1.8–8.2)	1 × 10^−3^	–	–	1.1 (0.4–3.2)	0.83		

PUV = posterior urethral valve; UPJO = ureteropelvic junction obstruction; SNP = single-nucleotide polymorphism; HR = hazard ratio; CI = confidence interval.

a These SNPs were imputed in part for the Dutch replication sample.

In an additional analysis, we adjusted the Cox regression analyses for rs6874819 among the discovery PUV patients for the possible prognostic factors from Table 1. Although 188 patients were excluded because of missing values for these factors, the analyses with 128 PUV patients with 47 events still gave statistically significant results very comparable to the unadjusted results for rs6874819 (hazard ratio for the GG genotype [HR_GG_] 23, *p* = 0.004; HR_GA_ 2.1, *p* = 0.03).

### 3.3 Replication analyses

Of the 222 Dutch replication samples, 78 were genotyped on the same platform as the discovery samples, while 144 were genotyped on the Global Screening array. Only two of the six suggestive SNPs (rs6874819 and rs2148707) were directly genotyped with the latter, so we used imputed genotypes for the others. We performed Kaplan-Meier and Cox regression analyses for 102 PUV and 120 UPJO patients, of whom 34 and 65, respectively, developed signs of kidney injury within 10 yr after surgery. Although not statistically significant, the associations of the SNPs on chromosome 5 were in the same direction as in the discovery sample for PUV patients (rs6874819: HR_GA_ 1.8, *p* = 0.11; HR_GG_ 2.4, *p* = 0.40; Fig. 2B). No associations were observed for the other SNPs in the PUV group or for the UPJO group. In the European replication sample of 102 PUV patients, the results were very similar, with a higher risk of kidney injury for carriers of variant genotypes on chromosome 5 (rs6874819: HR_GA_ 1.8, *p* = 0.11; HR_GG_ 3.1, *p* = 0.04; Table 2 and Fig. 2C).

### 3.4 Meta-analysis

We performed Cox regression analyses for the allelic effects in the discovery and replication samples, and subsequently performed a meta-analysis. This result was statistically significant at a genome-wide level, with a HR for the G allele of 2.3 (95% confidence interval 1.7–3.0; *p* = 4.1 × 10^–9^; Table 3).

**Table 3:** HRs for the allelic effect of the rs6874819 single-nucleotide polymorphism on kidney injury among patients with posterior urethral valves in the Dutch discovery, Dutch replication, and European replication samples, and results of the meta-analysis

Sample	Effect of G vs A allele	
	HR (95% CI)	*p* value
Dutch discovery	2.5 (1.7–3.7)	1 × 10^−6^
Dutch replication	1.7 (0.9–3.3)	8 × 10^−2^
European replication	2.2 (1.3–3.7)	3 × 10^−2^
Meta-analysis	2.3 (1.7–3.0)	4 × 10^−9^

HR = hazard ratio; CI = confidence interval.

### 3.5 Expression analyses

Visualization of the association results using imputed genotypes for the discovery sample in LocusZoom [22] showed that the peak on chromosome 5 was located in an intron of the *CDH12* gene (Supplementary Fig. 3). All studies in the Expression Atlas [17] revealed that *CDH12* shows the highest expression in human adult brain, whereas it is not expressed in adult kidney. The NIH Epigenomics Roadmap (http://www.roadmapepigenomics.org/) showed that *CDH12* is expressed in human fetal kidneys [23]. We confirmed that mRNA isolated from human adult kidney shows no *CDH12* expression, whereas adult brain tissue does. Fetal kidneys showed expression levels comparable to those in adult brain. Transcriptomic analyses of 15 human embryonic tissues and organs 33–55 d after conception revealed that *CDH12* is widely expressed in embryonic tissues, with highest levels in retinal pigmented epithelium, followed by the kidneys [18].

**Fig. 3:**
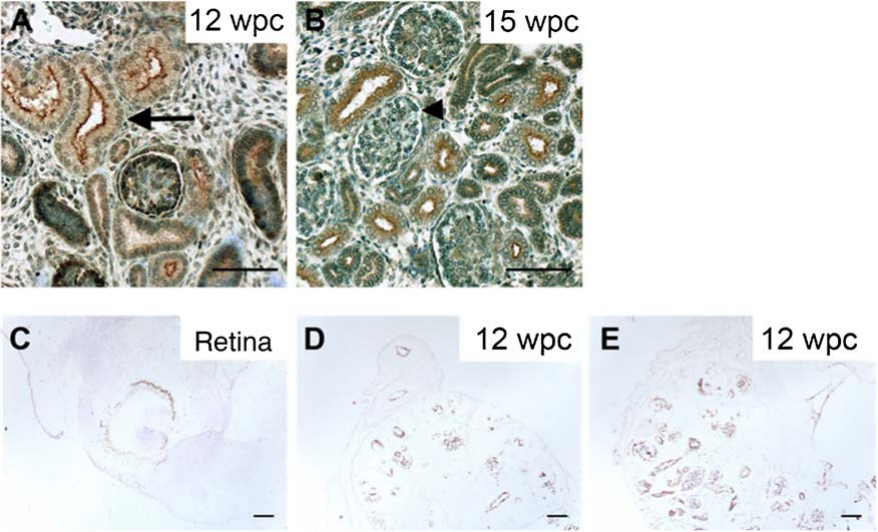
Immunohistochemistry profile for CDH12 in the developing human kidney. (A,B) Sagittal sections through the developing kidney. Brightfield images show CHD12 in brown, counterstained with toluidine blue. The arrow points to CHD12 within developing proximal tubules and the arrowhead indicates the developing glomerulus. Scale bars represent 50 μm. (C–E) Immunostaining results showing clear expression of CDH12 in (C) the retina of a 30-d-old mouse and (D,E) human kidneys 12 wk after conception (wpc). At approximately 12 wk after conception, CDH12 was expressed in the ureteric bud, the precursor of the collecting duct, and in early condensates, where the ureteric bud induces mesenchymal cells to condense and undergo mesenchymal-epithelial conversion, the first stage in nephron epithelial formation. Scale bars represent 100 μm. WPC stands for weeks post conception.

Immunohistochemical staining confirmed CDH12 expression in the fetal retina and kidney. At approximately 12 wk after conception, CDH12 was expressed in the ureteric bud, the precursor of the collecting duct, and in early condensates, where the ureteric bud induces mesenchymal cells to condense and start to form epithelia. In addition, CDH12 expression was localized to proximal tubules within the developing human kidney at 12 and 15 wk after conception (Fig. 3).

The GTEx portal showed that the associated area was an eQTL for *CDH12* in the adrenal gland. Several SNPs in the associated area resulted in higher expression of *CDH12* (Supplementary Figs. 4 and 5). Two of these SNPs were directly genotyped in our samples. The CC genotypes resulted in statistically significantly higher expression of *CDH12* in the adrenal gland according to the GTEx portal, and were associated with time to the development of signs of kidney injury in our data (Supplementary Table 3).

## 4 Discussion

We identified for the first time *CHD12* as a gene potentially involved in the development of kidney injury in patients with obstructive uropathies. This result was seen among PUV patients, but not clearly among UPJO patients, possibly because of the different timing of PUV and UPJO development, or because both kidneys are exposed to high urinary pressure in PUV, whereas generally only one kidney is in UPJO.

The association signal on chromosome 5 among PUV patients was the most clear and the other suggested SNPs were not associated with kidney injury in the Dutch replication sample. Detection of the signal for *CDH12* despite our small and nonhomogeneous sample suggests that it may be an important factor. This was confirmed by detection of the signal in the European PUV replication sample, which was a very different patient group. Although the primary results for rs6874819 were not statistically significant at a genome-wide level, the result for the allelic meta-analysis among PUV patients was. In addition, all results show the same direction of effect. The fact that our association signal is located in an eQTL and that CDH12 is expressed in fetal kidneys further enhances the reliability of our finding.

In an additional analysis, we adjusted our analyses in the European replication sample for eGFR at study entry, which changed the effect of rs6874819 only slightly. Since eGFR at study entry correlates with GFR at birth, this finding suggests that the effect of rs6874819 on kidney injury we observed is not sufficiently explained by effects of CHD12 on fetal kidney development. Our hypothesis is that the damage arises in the period when affected kidneys are exposed to elevated pressure, which is from early fetal development up to the postnatal period preceding surgery. The damage may not be severe enough to display signs of kidney injury at that point in time, but as the patients grow and need greater filtration capacity, the affected kidneys may fall short and the previously acquired damage begins to show.

*CDH12* is a member of the neural cadherin (N-cadherin) gene family, which encode transmembrane calcium-dependent homophilic adhesion receptors that play an important role in cell recognition and sorting during development [24]. *CDH12* has not been described in relation to kidney development before, but it is involved in the progression of several cancers [24], [25], [26], [27]. In addition, CDH12 may play a role in monocyte infiltration. Grandaliano et al [5] showed that the extent of monocyte infiltration in kidney tissue of children with UPJO correlated with the degree of tubulointerstitial damage, suggesting that monocyte infiltration is an important event in the development of kidney injury in obstructive uropathy. They also showed a striking increase in *MCP1* gene expression in these kidney tissues, which is in line with the suggestion by Diamond et al [28] that upregulation of Mcp1, in response to an increase in tubular pressure, stimulates monocyte influx in a rat model of UUO. Niu et al [29] found that MCP1 action in inducing angiogenesis is mediated via induction of a new transcription factor that they named MCP1-induced protein (MCPIP). Chromatin immunoprecipitation analysis showed that *CDH12* is a target of MCPIP [29]. Ma et al [27] confirmed that CDH12 expression is modulated by MCP1 in colorectal cancer cells.

A drawback of our study is the relatively small sample size. We may have missed less strong but still important associations because our discovery analyses only had enough power (80%) to detect variants with an allele frequency of 30% and genotype relative risk of 2.7 or higher under a multiplicative disease model. In addition, use of a more homogeneous cohort of, for example, only prenatally detected PUV, or a more homogeneous endpoint, such as the eGFR decrease in the first yr of life, may have been better. Instead, we used any sign of kidney injury as the outcome to achieve larger numbers, but did not have enough patients with specific signs to perform separate analyses for subgroups. Nevertheless, when we performed additional analyses that excluded one kidney functioning at <45% from the definition of signs of kidney injury (the diagnosis that explains why more UPJO than PUV patients have signs of kidney injury), results for PUV patients in the discovery sample were very comparable to the initial results. Using a more homogeneous cohort would result in patients being more comparable for other factors that could influence the prognosis of PUV [30]. However, when we adjusted the Cox regression analyses for PUV patients of the discovery sample for the possible prognostic factors from Table 1, the results were very comparable to the unadjusted results for rs6874819. This suggests that the effect of rs6874819 is independent from these factors and that it may influence the risk of signs of kidney injury in all PUV patients, regardless of severity or management.

## 5 Conclusions

We hypothesize that MCP1 pathway upregulation due to an increase in urinary tract pressure in PUV patients is augmented in carriers of the *CDH12* variant, which facilitates monocyte infiltration and the development of progressive kidney fibrosis and exacerbates kidney injury. This hypothesis is in line with the importance of monocyte infiltration in the development of kidney injury in obstructive uropathy and provides leads for further research. The role of *CDH12* in monocyte infiltration and the development of kidney injury in PUV, and the predictive value of rs6874819 for the prognosis of individual PUV patients are important future research topics, as is the generalizability of our findings to other forms of obstructive uropathy.

  ***Author contributions:*** Loes F.M. van der Zanden had full access to all the data in the study and takes responsibility for the integrity of the data and the accuracy of the data analysis.

  *Study concept and design:* van der Zanden, van Rooij, Renkema, Schreuder, Roeleveld, Feitz.

*Acquisition of data:* van der Zanden, van Rooij, de Wall, Bongers, Renkema, Schreuder, Roeleveld, Feitz, Quaedackers, Nijman, Steffens, Schaefer, Kirchner, Behnisch, Bayazit, Caliskan, Obrycki, Montini, Duzova.

*Analysis and interpretation of data*: van der Zanden, van Rooij, Renkema, Schreuder, Roeleveld, Feitz, Schaefer, Kirchner, Behnisch, Wuttke, Jennings, Hanley, Milmoe, Winyard.

*Drafting of the manuscript:* van der Zanden.

*Critical revision of the manuscript for important intellectual content:* van Rooij, Quaedackers, Nijman, Steffens, de Wall, Bongers, Schaefer, Kirchner, Behnisch, Bayazit, Caliskan, Obrycki, Montini, Duzova, Wuttke, Jennings, Hanley, Milmoe, Winyard, Renkema, Schreuder, Roeleveld, Feitz.

*Statistical analysis:* van der Zanden, Behnisch, Hanley.

*Obtaining funding:* van der Zanden.

*Administrative, technical, or material support:* None.

*Supervision:* Feitz, Roeleveld, Schreuder.

*Other:* None.

  ***Financial disclosures:*** Loes F.M. van der Zanden certifies that all conflicts of interest, including specific financial interests and relationships and affiliations relevant to the subject matter or materials discussed in the manuscript (eg, employment/affiliation, grants or funding, consultancies, honoraria, stock ownership or options, expert testimony, royalties, or patents filed, received, or pending), are the following: Rien J.M. Nijman has received lecture fees from AstraZeneca, Giovanni Montini has received consultancy fees from Merck, and Neil A. Hanley has received consultancy fees from APIS Assay Technologies, all outside the scope of the current manuscript. The remaining authors have nothing to disclose.

  ***Funding/Support and role of the sponsor:*** This research was supported by a Kolff junior postdoctoral grant from the 10.13039/501100002997Dutch Kidney Foundation (13OKJ36) and a ZonMW-VENI grant from The 10.13039/501100003246Netherlands Organisation for Scientific Research (91618036). The sponsors played a role in the design and conduct of the study.

  ***Acknowledgments:*** We would like to thank all those involved in data collection, all patients and parents for their cooperation in this study, and deCODE Genetics (Reykjavik, Iceland) for performing the microarray experiments. Part of the collaboration was supported by the European Reference Networks (ERNs) for rare urogenital diseases and complex condition (ERN eUROGEN) and for Rare Kidney Diseases (ERKNet). Fetal tissue was provided at the UCL GOS Institute of Child Health by the MRC-Wellcome Trust Human Developmental Biology Resource.

  ***Ethics considerations:*** This study was approved by the Regional Committee on Research Involving Human Subjects Arnhem-Nijmegen and by the board of directors of the Isala Clinics and University Medical Center, Groningen. The 4C study was approved by the central ethics committee of Heidelberg University Medical Faculty and by each local institutional review board. All participants and/or their parents provided written informed consent. The collection, storage, and use of human embryonic and fetal material were approved by the North West Research Ethics Committee or via the Human Developmental Biology Resource, under the codes of practice issued by the Human Tissue Authority and legislation of the UK Human Tissue Act 2008.
