# Supplementary material for: CDH12 as a Candidate Gene for Kidney Injury in Posterior Urethral Valve Cases: A Genome-wide Association Study Among Patients with Obstructive Uropathies
Source: Eur Urol Open Sci. 2021 Apr 24;28:26–35. doi: 10.1016/j.euros.2021.04.001 (PMC8317879; doi:10.1016/j.euros.2021.04.001)
Supplement: Supplementary file 1 [file mmc1.docx]

**Supplemental Material**

***CDH12* candidate gene for kidney injury in posterior urethral valves;**

**A genome-wide association study among patients with obstructive uropathies.**

Loes F.M. van der Zanden, PhD^1,*^, Iris A.L.M. van Rooij, PhD^1^, Josine S.L.T. Quaedackers, MD, PhD^2^, Rien Nijman, Prof.^2^, Martijn Steffens, MD, PhD^3^, Liesbeth L.L. de Wall, MD^4^, Ernie M.H.F. Bongers, MD, PhD^5^, Franz Schaefer, Prof^6^, Marietta Kirchner, PhD^7^, Rouven Behnisch, MSc^7^, Aysun K. Bayazit, Prof^8^, Salim Caliskan, Prof^9^, Lukasz Obrycki, MD, PhD^10^, Giovanni Montini, Prof^11,12^, Ali Duzova, Prof^13^, Matthias Wuttke, MD, PhD^14^, Rachel Jennings, MD, PhD^15,16^, Neil A. Hanley, Prof, ^15,16^, Natalie J. Milmoe, MSc^17^, Paul J.D. Winyard, Prof^17^, Kirsten Y. Renkema, PhD^18^, Michiel F. Schreuder, MD, PhD^19,&^, Nel Roeleveld, PhD^1,&^, Wout F.J. Feitz, Prof^4,&^.

*^*^ Corresponding author; ^&^ Shared last authorship*

*1 Radboud Institute for Health Sciences, Department for Health Evidence, Radboud university medical center, Nijmegen, The Netherlands 2 Department of Urology, University Medical Center Groningen, Groningen, The Netherlands*

*3 Department of Urology, Isala, Zwolle, The Netherlands*

*4 Radboud Institute for Molecular Life Sciences, Division of Pediatric Urology, Department of Urology, Radboudumc Amalia Children’s Hospital, Nijmegen, The Netherlands*

*5 Radboud Institute for Molecular Life Sciences, Department of Human Genetics, Radboud university medical center, Nijmegen, The Netherlands*

*6 Center for Pediatrics and Adolescent Medicine, University Hospital Heidelberg, Heidelberg, Germany*

*7 Institute of Medical Biometry and Informatics, University of Heidelberg, Germany*

*8 Department of Pediatric Nephrology, Cukurova University, Faculty of Medicine, Adana, Turkey*

*9 Department of Pediatric Nephrology, Istanbul University-Cerrahpasa, Turkey*

*10 Department of Nephrology, Kidney Transplantation and Hypertension, Children`s Memorial Health Institute, Warsaw, Poland*

*11 Pediatric Nephrology, Dialysis and Transplant Unit, Fondazione IRCCS Ca’ Granda – Ospedale Maggiore Policlinico di Milano, Milan, Italy*

*12 Department of Clinical Sciences and Community Health, University of Milan, Italy*

*13* *Division of Pediatric Nephrology, Hacettepe University Faculty of Medicine, Ankara, Turkey*

*14 Institute of Genetic Epidemiology, Faculty of Medicine and Medical Center, University of Freiburg, Freiburg, Germany*

*15 Faculty of Biology, Medicine & Health, Manchester Academic Health Sciences Centre, University of Manchester, Manchester, UK*

*16 Endocrinology Department, Manchester University NHS Foundation Trust, Manchester, UK*

*17 Nephro-Urology, Developmental Biology and Cancer Programme, UCL Great Ormond Street Institute of Child Health, London, UK*

*18 Department of Genetics, Center for Molecular Medicine, University Medical Center Utrecht, Utrecht University, Utrecht, The Netherlands*

*19 Radboud Institute for Molecular Life Sciences, Department of Pediatric Nephrology, Radboudumc Amalia Children’s Hospital, Nijmegen, The Netherlands*

**TABLE OF CONTENTS**

**Supplementary methods** 3

Description of the definition of signs of kidney injury

Discovery and Dutch replication sample 3

European replication sample 4

Description of genotyping and quality control

Discovery and Dutch replication sample 5

European replication sample 6

Description of imputation 6

**Supplementary figures** 7

**Supplementary tables** 12

**References** 15

**SUPPLEMENTARY METHODS**

**Description of the definition of signs of kidney injury**

*Discovery and Dutch replication sample*

Medical files of the Dutch patients were scrutinized to obtain information about kidney function. Patients were defined as having signs of kidney injury in case of dialysis, nephrectomy, kidney transplantation, eGFR<60 ml/min/1.73m^2^, high blood pressure, antihypertensive medication use, proteinuria, and/or one kidney functioning<45%.

To determine eGFR, we used the Bedside Schwartz formula (eGFR=0.413×(height (cm)/serum creatinine (mg/dl)) for children between 3 months and 18 years of age. If height at the time of creatinine measurement was not known, we extrapolated the height percentile measured at another moment in time. If no height measurement was available, we assumed the child to be at the 50^th^ percentile for age and sex. For persons aged 18 years or older, we used the CKD-EPI formula to determine eGFR.

To determine whether children had a high blood pressure, we used the reference blood pressures from the Fourth Report on the diagnosis of high blood pressure in children,^1^ taking into account gender, age and height. If height at the time of blood pressure measurement was unknown, we followed the same procedure as described above for eGFR. Children with a systolic and/or diastolic blood pressure>95^th^ percentile were defined as having high blood pressure. For patients aged 16 years or older, we used a systolic blood pressure>140mmHg and/or a diastolic blood pressure>90mmHg as high blood pressure. A high blood pressure was not considered as a sign of kidney injury if the high blood pressure was likely to have another cause (e.g. in children with coarctation of the aortae, tetralogy of Fallot, transposition of the great arteries, neurofibromatosis, and hypertrophic cardiomyopathy). Patients using antihypertensive medication were also considered as having signs of kidney injury, unless the medication was given for other reasons than high blood pressure (e.g. thiazides for hypercalciuria).

Proteinuria was defined as a urinary protein-to-creatinine ratio >0.55g/10mmol for children aged 6 to 24 months, and >0.22g/10mmol for patients aged 2 years or older. Lastly, patients who showed that one kidney was functioning for <45% on MAG3 renography or DMSA scan were defined as having signs of kidney injury, unless the patient already had a solitary functioning kidney before surgery. If multiple MAG3 scans were performed, we used the results of the most recent scan.

To determine follow-up time, we used the last date that a patient visited the hospital or the hospital received a letter from another physician. The questionnaires that parents filled out about signs of kidney injury was not used to determine the occurrence of signs of kidney injury, because we considered the medical files to be more accurate. However, if both the questionnaire and the medical file indicated no signs of kidney injury, we used the date of filling out the questionnaire as last follow-up date.

*European replication sample*

The primary endpoint was kidney injury defined as a composite of 50% loss of eGFR that persisted for at least one month, the start of kidney replacement therapy, or an eGFR less than 10 mL/min/1.73 m², whichever occurred first. If a 50% reduction in eGFR occurred between two study visits, interpolation was used to determine the time of event. eGFR values for evaluating kidney survival were calculated using the formula of Schwartz et al.^2^ incorporating patient height, serum creatinine, serum cystatin C, and blood urea nitrogen.

**Description of genotyping and quality control**

*Discovery and Dutch replication sample*

DNA samples were isolated from blood or saliva using standard methods in a certified laboratory. Subsequently, the DNA samples were diluted to 100 ng/µl, the absorbance ratio 260/280 was measured as an indicator of protein contamination, and samples were separated by size on gel to visualize DNA degradation. Subsequently, DNA samples were used in the genotyping analyses.

The discovery samples were genotyped by deCODE Genetics (Reykjavik, Iceland) using Infinium OmniExpress bead chips (Illumina, San Diego, CA, USA). This resulted in 705,269 genotyped SNPs. Variants were excluded if they had<95% yield (N=7,079), differed in genotyping rate between cases and controls (P<1×10^-4^, N=0), had minor allele frequency (MAF)<1% (N=75,438), or failed the Hardy Weinberg Equilibrium (HWE) test (P<1×10^−4^, N=55), resulting in 622,697 variants for the discovery analyses.

The Dutch replication samples were genotyped using Infinium OmniExpress bead chips (n=78) or Global Screening arrays (n=144) (Illumina). These microarrays contained 176,825 overlapping SNPs, of which the 172,976 variants that survived SNP quality control (N=1,118 <95% yield, N=2,712 MAF<1%, N=14 failed HWE, N=5 different genotyping rate between chips (P<1×10^-4^)) were used in the sample quality control steps. Genotype data are available upon request.

In our sample quality control, we excluded samples with a call rate<97%, discordant sex information, or relatedness (π-hat)>0.1875. In the identification of ethnic outliers, PLINK was used to compute principal components (PC) for all participants and reference samples of the HapMap project Phase 3.^3^ Visualization of sub-structuring was done by the multi-dimensional scaling approach in PLINK. Participants with PC1 or PC2 values more than three standard deviations from the mean of the known Northern European ancestry group were excluded. We repeated PC analyses in remaining participants, visualized results, and excluded remaining ethnic outliers.

*European replication sample*

European replication samples were genotyped using Illumina Infinium 2.5M-8 microarrays. Quality control steps were performed according to standard protocols. SNPs with call rate<95% were removed, yielding an average of 1.7 million markers.

**Description of imputation**

Genotypes of the Dutch discovery and replication samples were phased using SHAPEIT (v2.790)^4^ and imputed using IMPUTE2 (v2.3.2)^5^ with The Genome of the Netherlands (GoNL) dataset of 249 Dutch trios as reference panel.^6^ Imputation was performed separately for samples genotyped on either array. For the European replication sample, imputation was performed using SHAPEIT (v2.r644) and IMPUTE2 (v2.3.0) with phased haplotypes from the 1000 Genomes project (phase 1, release v3 date 2012-04-19, ALL subset) as reference panel. After imputation, ~10.5 million SNPs of high imputation quality (info measure ≥0.8) were retained.

**SUPPLEMENTARY FIGURES**

**
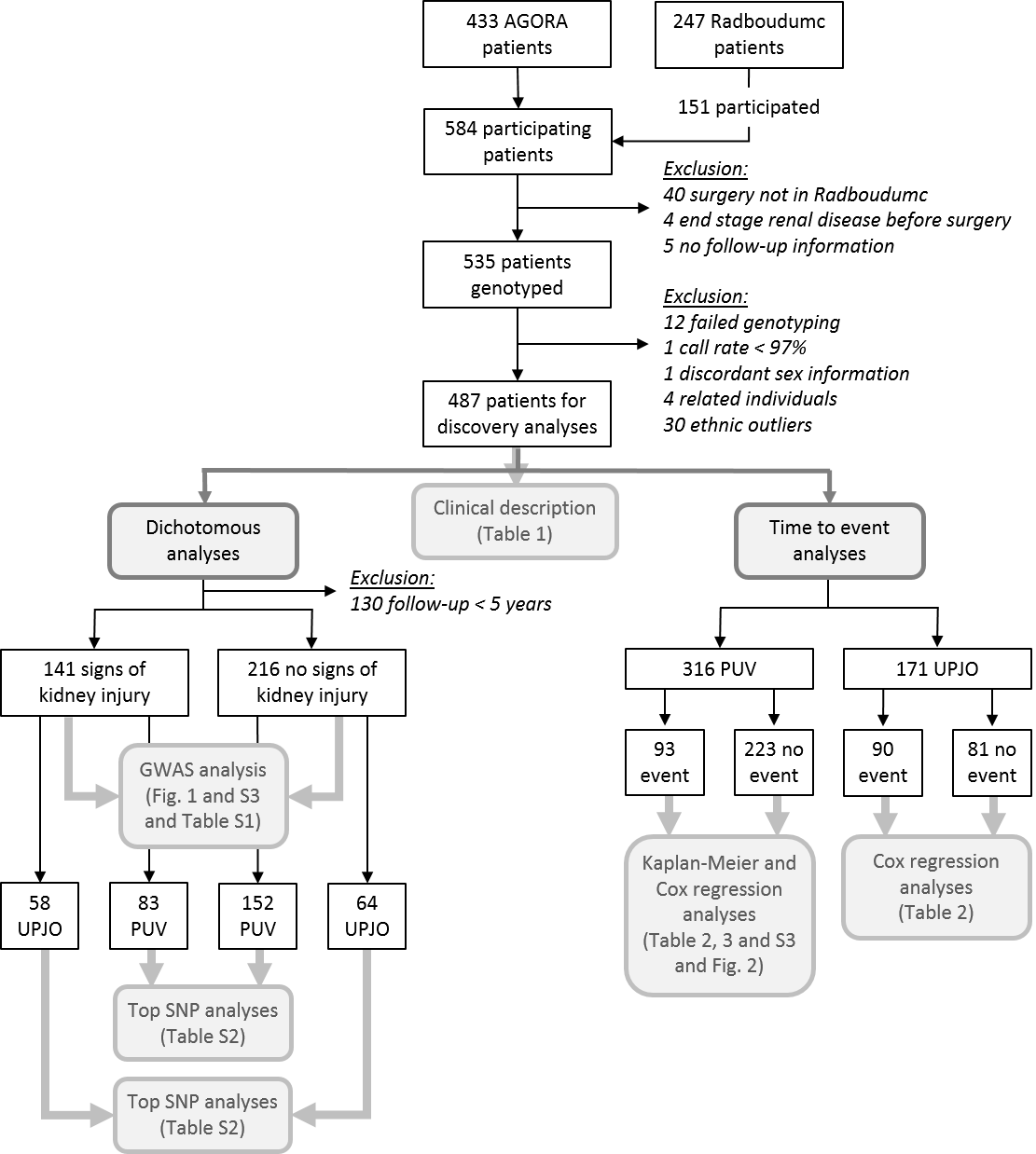
**

***Fig S1*** *Flowchart showing patient in- and exclusion for the different analyses in the discovery*

*sample.*

***
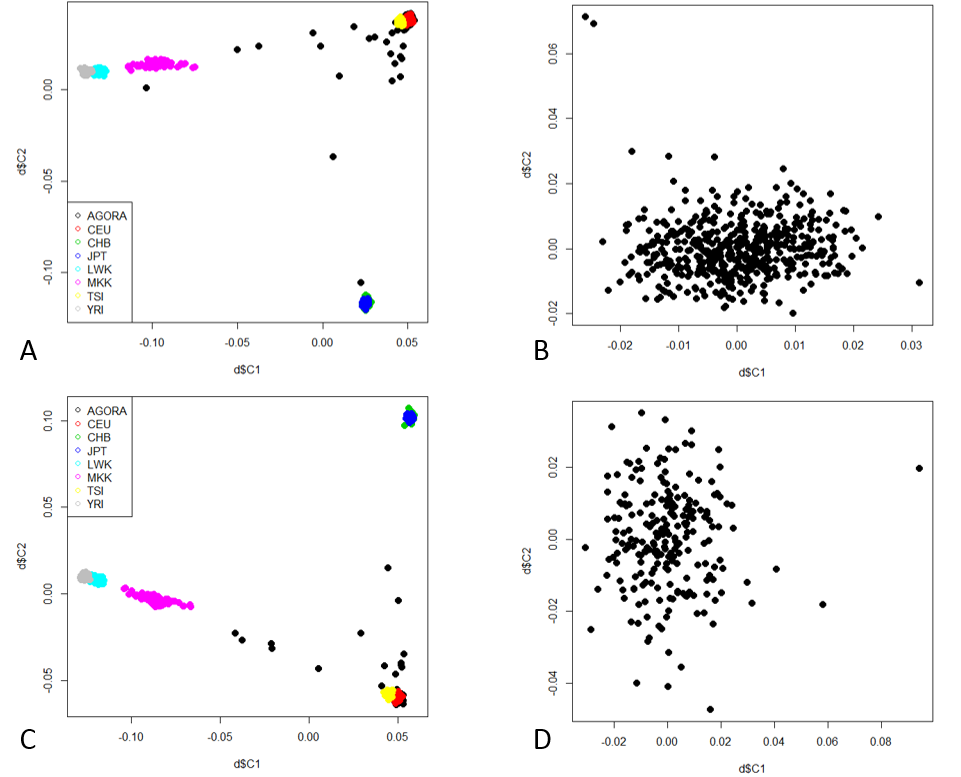
***

***Fig S2*** *Visualization of sub-structuring by the multi-dimensional scaling approach implemented in PLINK (A) in the Dutch discovery and HapMap samples, after exclusion of the ASW (African ancestry in Southwest USA), CHD (Chinese in Metropolitan Denver, Colorado), GIH (Gujarati Indians in Houston, Texas) and MXL (Mexican ancestry in Los Angeles, California) populations, (B) in the Dutch discovery samples after exclusion of the 28 individuals with PC1 or PC2 values more than 3 sd from the mean of the known Northern European ancestry group, (C) in the Dutch replication and HapMap samples, after exclusion of the ASW, CHD, GIH and MXL populations, and (D) in the Dutch replication samples after exclusion of the 23 individuals with PC1 or PC2 values more than 3 sd from the mean of the known Northern European ancestry group. Plot A and C show that samples from the same population cluster, as do most samples from the Dutch discovery and replication sample. Plot B and D show that, after exclusion of the individuals with PC1 or PC2 values more than 3 sd from the mean of the known Northern European ancestry group, there are 2 remaining outliers in the discovery sample and no remaining outliers in the Dutch replication sample. AGORA; Dutch discovery or replication samples, CEU; Utah residents with Northern and Western European ancestry from the CEPH collection, CHB; Han Chinese in Beijing, China, JPT; Japanese in Tokyo, Japan, LWK; Luhya in Webuye, Kenya, MKK; Maasai in Kinyawa, Kenya, TSI; Toscani in Italia, YRI; Yoruba in Ibadan, Nigeria.*


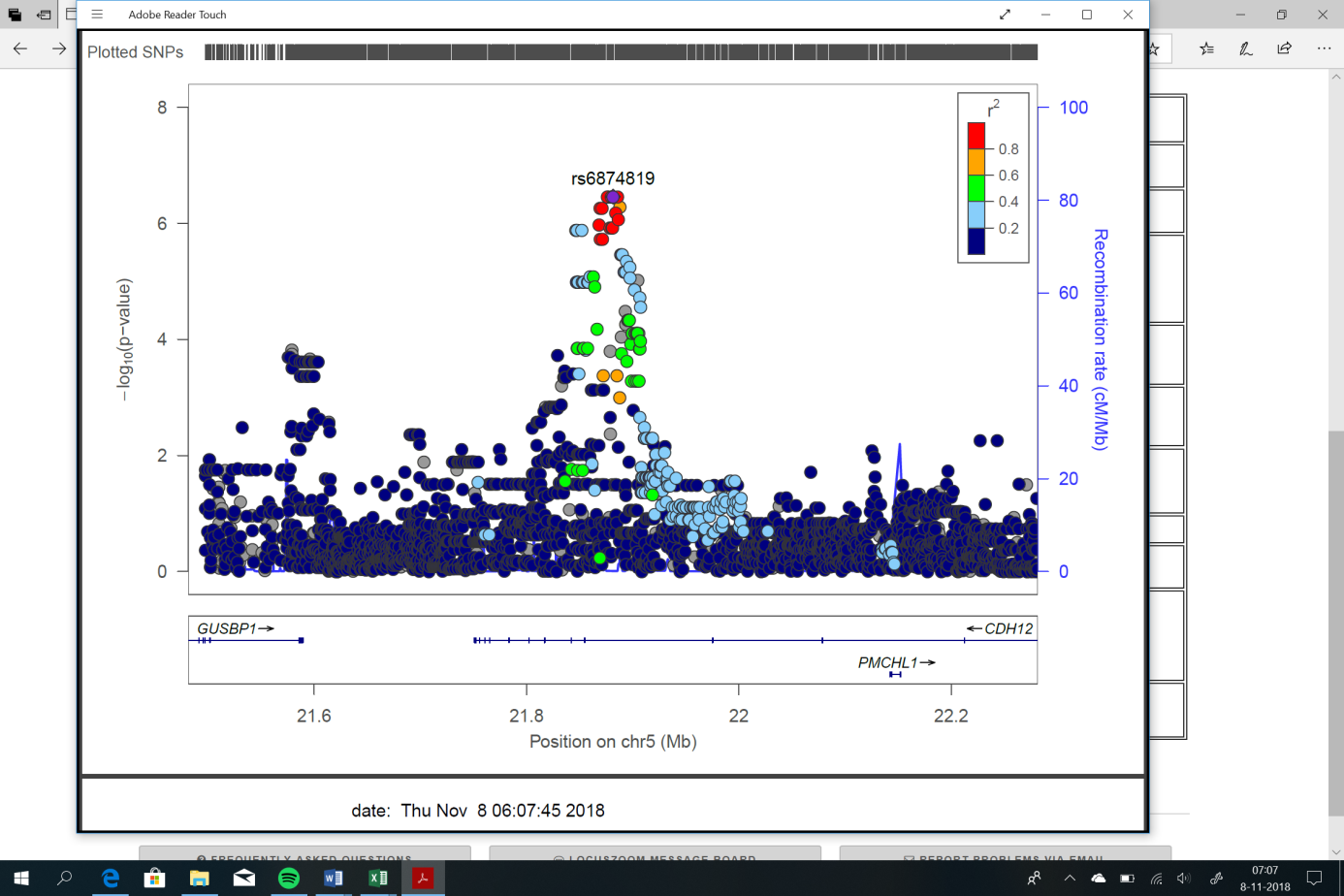


***Fig S3*** *Visualization of association signal on chromosome 5 using imputed genotypes of the discovery sample in LocusZoom.*

***
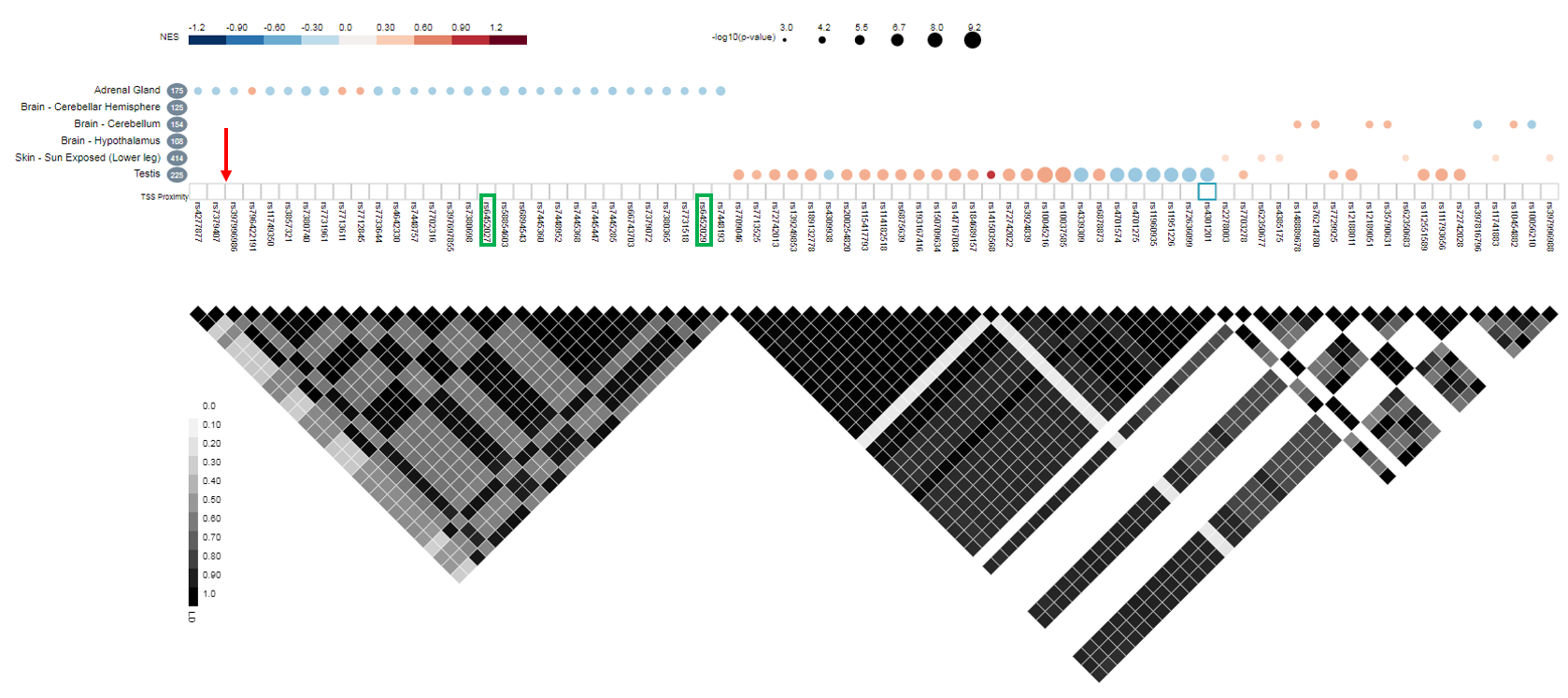
***

***Fig S4*** *Figure from the GTEx portal showing significant cis-eQTLs in the CDH12 gene. The red arrow shows the location of rs6874819, which is not displayed in the GTEx portal. This figure shows that rs6452027 and rs6452029 (the eQTL SNPs within the green frames, which were directly genotyped in the discovery and replication samples) result in less expression of CDH12. In the GTEx portal, however, the CC genotypes (which are least frequent) are used as reference genotypes (see Fig. S5). These genotypes are our risk genotypes (see Table S3), which means that our risk genotypes result in higher CDH12 expression.*

A***
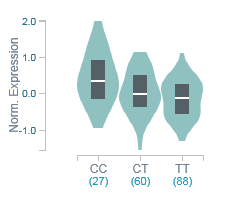
***B *
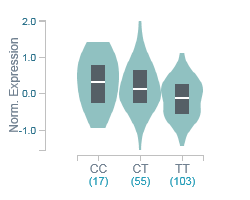
*

***Fig S5*** *Violin plots from the GTEx portal showing differential expression patterns for the genotypes of (A) rs6452027 and (B) rs6452029.*

**SUPPLEMENTARY TABLES**

***Table S1*** *Association results for kidney injury for the genotyped SNPs passing the*

*1×10^-5^ threshold in patients with obstructive uropathy.*

| **CHR** | **Gene** | **BP** | **SNP** | **Genotype** | **Controls**  **N=216 (%)** | **Cases**  **N=141 (%)** | **OR (95%CI)** | **OR_adj_** | **P_adj_** |
| --- | --- | --- | --- | --- | --- | --- | --- | --- | --- |
| 5 | *CDH12* | 21881405 | rs6874819 | A | 408 (94) | 233 (83) | ref | ref |  |
|  |  |  |  | G | 24 (6) | 49 (17) | 3.6 (2.1-6.0) | 4.0 | 7.5E^-7^ |
|  |  |  |  | AA | 192 (89) | 94 (67) | ref |  |  |
|  |  |  |  | GA | 24 (11) | 45 (32) | 3.8 (2.2-6.7) |  |  |
|  |  |  |  | GG | 0 (0) | 2 (1) | - |  |  |
|  |  | 21880907 | rs9292998 | A | 407 (95) | 236 (84) | ref | ref |  |
|  |  |  |  | G | 23 (5) | 46 (16) | 3.4 (2.0-5.8) | 3.7 | 3.1E^-6^ |
|  |  |  |  | AA | 192 (89) | 97 (69) | ref |  |  |
|  |  |  |  | GA | 23 (11) | 42 (30) | 3.6 (2.1-6.4) |  |  |
|  |  |  |  | GG | 0 (0) | 2 (1) | - |  |  |
|  |  | 21847422 | rs12171538 | T | 374 (87) | 203 (72) | ref | ref |  |
|  |  |  |  | C | 58 (13) | 79 (28) | 2.5 (1.7-3.7) | 2.4 | 7.1E^-6^ |
|  |  |  |  | TT | 162 (75) | 75 (53) | ref |  |  |
|  |  |  |  | CT | 50 (23) | 53 (38) | 2.3 (1.4-3.7) |  |  |
|  |  |  |  | CC | 4 (2) | 13 (9) | 7.0 (2.2-22) |  |  |
| 8 | *DLGAP2* | 1618339 | rs2957086 | A | 257 (59) | 120 (43) | ref | ref |  |
|  | *DLGAP2-AS1* |  |  | G | 175 (41) | 162 (57) | 2.0 (1.5-2.7) | 2.1 | 9.3E^-6^ |
|  |  |  |  | AA | 69 (32) | 30 (21) | ref |  |  |
|  |  |  |  | GA | 119 (55) | 60 (43) | 1.2 (0.7-2.0) |  |  |
|  |  |  |  | GG | 28 (13) | 51 (36) | 4.2 (2.2-7.9) |  |  |
| 13 | 10kB down- | 21224358 | rs9580025 | C | 359 (83) | 193 (68) | ref | ref |  |
|  | stream of |  |  | T | 73 (17) | 89 (32) | 2.3 (1.6-3.2) | 2.4 | 8.2E^-6^ |
|  | *LINC01046* |  |  | CC | 147 (68) | 66 (47) | ref |  |  |
|  |  |  |  | TC | 65 (30) | 61 (43) | 2.1 (1.3-3.3) |  |  |
|  |  |  |  | TT | 4 (2) | 14 (10) | 7.8 (2.5-25) |  |  |
|  |  | 21225186 | rs2148707 | G | 359 (83) | 193 (68) | ref | ref |  |
|  |  |  |  | T | 73 (17) | 89 (32) | 2.3 (1.6-3.2) | 2.4 | 8.2E^-6^ |
|  |  |  |  | GG | 147 (68) | 66 (47) | ref |  |  |
|  |  |  |  | TG | 65 (30) | 61 (43) | 2.1 (1.3-3.3) |  |  |
|  |  |  |  | TT | 4 (2) | 14 (10) | 7.8 (2.5-25) |  |  |

*CHR, chromosome; BP, basepair; SNP, single nucleotide polymorphism; N, number; OR, odds ratio; CI, confidence interval; OR_adj_, odds ratio adjusted for first 4 principal components (only for allelic tests); P_adj_, P-value adjusted for first 4 principal components (only for allelic tests); kB, kilobases.*

***Table S2*** *Association results for kidney injury for the genotyped SNPs passing the*

*1×10^-5^ threshold separately for PUV and UPJO patients.*

|  |  |  | **PUV patients** | | | **UPJO patients** | | |
| --- | --- | --- | --- | --- | --- | --- | --- | --- |
| **CHR** | **SNP** | **Geno-**  **type** | **Controls**  **N=152 (%)** | **Cases**  **N=83 (%)** | **OR (95%CI)** | **Controls**  **N=64 (%)** | **Cases**  **N=58 (%)** | **OR (95%CI)** |
| 5 | rs6874819 | A | 287 (94) | 134 (81) | ref | 121 (95) | 99 (85) | ref |
|  |  | G | 17 (6) | 32 (19) | 4.0 (2.2-7.5) | 7 (5) | 17 (15) | 3.0 (1.2-7.4) |
|  |  | AA | 135 (89) | 52 (63) | ref | 57 (89) | 42 (72) | ref |
|  |  | GA | 17 (11) | 30 (36) | 4.6 (2.3-9.0) | 7 (11) | 15 (26) | 2.9 (1.1-7.8) |
|  |  | GG | 0 (0) | 1 (1) | - | 0 (0) | 1 (2) | - |
|  | rs9292998 | A | 285 (94) | 134 (81) | ref | 122 (95) | 102 (88) | ref |
|  |  | G | 17 (6) | 32 (19) | 4.0 (2.1-7.5) | 6 (5) | 14 (12) | 2.8 (1.0-7.5) |
|  |  | AA | 134 (89) | 52 (63) | ref | 58 (91) | 45 (78) | ref |
|  |  | GA | 17 (11) | 30 (36) | 4.5 (2.3-8.9) | 6 (9) | 12 (12) | 2.6 (0.9-7.4) |
|  |  | GG | 0 (0) | 1 (1) | - | 0 (0) | 1 (2) | - |
|  | rs12171538 | T | 263 (87) | 111 (67) | ref | 111 (87) | 92 (79) | ref |
|  |  | C | 41 (13) | 55 (33) | 3.2 (2.0-5.0) | 17 (13) | 24 (21) | 1.7 (0.9-3.4) |
|  |  | TT | 113 (74) | 38 (46) | ref | 49 (91) | 37 (64) | ref |
|  |  | CT | 37 (24) | 35 (42) | 2.8 (1.6-5.1) | 13 (20) | 18 (31) | 1.8 (0.8-4.2) |
|  |  | CC | 2 (1) | 10 (12) | 15 (3.1-71) | 2 (3) | 3 (5) | 2.0 (0.3-13) |
| 8 | rs2957086 | A | 177 (58) | 72 (43) | ref | 80 (63) | 48 (41) | ref |
|  |  | G | 127 (42) | 94 (57) | 1.8 (1.2-2.7) | 48 (38) | 68 (59) | 2.4 (1.4-3.9) |
|  |  | AA | 45 (30) | 18 (22) | ref | 24 (38) | 12 (21) | ref |
|  |  | GA | 87 (57) | 36 (43) | 1.0 (0.5-2.0) | 32 (50) | 24 (41) | 1.5 (0.6-3.6) |
|  |  | GG | 20 (13) | 29 (35) | 3.6 (1.6-8.0) | 8 (13) | 22 (38) | 5.5 (1.9-16) |
| 13 | rs9580025 | C | 254 (84) | 110 (66) | ref | 105 (82) | 83 (72) | ref |
|  |  | T | 50 (16) | 56 (34) | 2.6 (1.7-4.0) | 23 (18) | 33 (28) | 1.8 (1.0-3.3) |
|  |  | CC | 106 (70) | 36 (43) | ref | 41 (64) | 30 (52) | ref |
|  |  | TC | 42 (28) | 38 (46) | 2.7 (1.5-4.8) | 23 (36) | 23 (40) | 1.4 (0.6-2.9) |
|  |  | TT | 4 (3) | 9 (11) | 6.6 (1.9-23) | 0 (0) | 5 (9) | - |
|  | rs2148707 | G | 254 (84) | 110 (66) | ref | 105 (82) | 83 (72) | ref |
|  |  | T | 50 (16) | 56 (34) | 2.6 (1.7-4.0) | 23 (18) | 33 (28) | 1.8 (1.0-3.3) |
|  |  | GG | 106 (70) | 36 (43) | ref | 41 (64) | 30 (52) | ref |
|  |  | TG | 42 (28) | 38 (46) | 2.7 (1.5-4.8) | 23 (36) | 23 (40) | 1.4 (0.6-2.9) |
|  |  | TT | 4 (3) | 9 (11) | 6.6 (1.9-23) | 0 (0) | 5 (9) | - |

*PUV, posterior urethral valves; UPJO, uteropelvic junction obstruction; CHR, chromosome; SNP, single nucleotide polymorphism; N, number; OR, odds ratio; CI, confidence interval.*

***Table S3*** *Hazard ratios for kidney injury in patients with posterior urethral valves for the eQTL SNPs that were directly genotyped in the Dutch discovery sample and in the Dutch and European replication samples.*

|  |  |  | ***Dutch discovery***  ***N = 316 (93 events)*** | | ***Dutch replication***  ***N=102 (34 events)*** | | ***European replication***  ***N=102 (54 events)*** | |
| --- | --- | --- | --- | --- | --- | --- | --- | --- |
| *CHR* | *SNP* | *Geno-*  *type* | *HR (95%CI)* | *P* | *HR (95%CI)* | *P* | *HR (95%CI)* | *P* |
| 5 | rs6452027 | TT | ref |  | ref |  | ref |  |
|  |  | CT | 2.1 (1.4-3.2) | 1E^-3^ | 1.6 (0.8-3.2) | 0.18 | 1.2 (0.7-2.3) | 0.50 |
|  |  | CC | 4.8 (2.3-9.8) | 2E^-5^ | 1.0 (0.1-7.5) | 1.00 | 3.6 (1.5-8.6) | 4E^-3^ |
|  | rs6452029 | TT | ref |  | ref |  | ref |  |
|  |  | CT | 2.4 (1.6-3.6) | 5E^-5^ | 1.6 (0.8-3.2) | 0.20 | 1.4 (0.7-2.7) | 0.20 |
|  |  | CC | 2.5 (0.6-10) | 0.21 | 2.4 (0.3-18) | 0.40 | 2.4 (0.9-6.4) | 0.07 |

*CHR, chromosome; SNP, single nucleotide polymorphism; HR, hazard ratio; CI, confidence interval; P, p-value.*

**REFERENCES**

1. National High Blood Pressure Education Program Working Group on High Blood Pressure in Adolescents. The fourth report on the diagnosis, evaluation, and treatment of high blood pressure in children and adolescents. Pediatrics. 2004;114:555-76.

2. Schwartz GJ, Munoz A, Schneider MF, et al. New equations to estimate GFR in children with CKD. JASN 2009;20:629-637.

3. International HapMap Consortium, Altshuler DM, Gibbs RA, et al. Integrating common and rare genetic variation in diverse human populations. Nature. 2010;467:52-8.

4. Delaneau O, Howie B, Cox AJ, et al. Haplotype estimation using sequencing reads. Am J Hum Genet. 2013;93:687-96.

5. Howie BN, Donnelly P, Marchini J. A flexible and accurate genotype imputation method for the next generation of genome-wide association studies. PLoS genetics. 2009;5:e1000529.

6. Genome of the Netherlands. Whole-genome sequence variation, population structure and demographic history of the Dutch population. Nature genetics. 2014;46:818-25.
